# Test-retest analysis of a non-invasive method of quantifying [^11^C]-PBR28 binding in Alzheimer’s disease

**DOI:** 10.1186/s13550-016-0226-3

**Published:** 2016-09-27

**Authors:** Akshay Nair, Mattia Veronese, Xiaohui Xu, Charles Curtis, Federico Turkheimer, Robert Howard, Suzanne Reeves

**Affiliations:** 1 UCL Huntington’s Disease Research Group, 2nd floor, Russell Square House, 10-12 Russell Square, London, WC1B 5EH UK; 2 Centre for Neuroimaging Sciences, IoPPN, King’s College London, Box PO89, De Crespigny Park, London, SE5 8AF UK; 3 MRC Social, Genetic and Developmental Centre, Institute of Psychiatry, Psychology & Neuroscience, King’s College London, De Crespigny Park, Denmark Hill, London, SE5 8EF UK; 4 NIHR Biomedical Research Centre for Mental Health, Maudsley Hospital and Institute of Psychiatry, Psychology & Neuroscience, King’s College London, London, SE5 8AF UK; 5 Division of Psychiatry, University College London, 6th Floor, Wings A and B, Maple House, 149 Tottenham Court Road, London, W1T 7NF UK

**Keywords:** Alzheimer’s, Dementia, Microglia, Inflammation, Positron emission tomography

## Abstract

**Purpose:**

In order to maximise the utility of [^11^C]-PBR28 for use in longitudinal studies and clinical trials in Alzheimer’s disease (AD), there is a need to develop non-invasive metrics of tracer binding that do not require arterial cannulation. Recent work has suggested that standardised uptake value (SUV)-based methods may be sensitive to changes in translocator protein (TSPO) levels associated with neurodegeneration. However, the test-retest reliability of these approaches in AD over a time period relevant for clinical trials is unknown. In this study, the test-retest reliability of three SUV-based metrics was assessed in AD patients over 12 weeks.

**Methods:**

Five patients with mild AD and the high-affinity binding TSPO genotype underwent two [^11^C]-PBR28 PET scans approximately 12 weeks apart. The test-retest reliability (TRR) of the unadjusted SUV, SUV relative to cerebellar grey matter (SUVR_C_) and SUV normalised to whole brain activity (SUVR_WB_) in nine cortical and limbic regions of interest was assessed using the absolute variability and the intraclass correlation coefficient.

**Results:**

Of the three measures, SUVR_WB_ performed best overall, showing low absolute variability (mean −0.13 %, SD 2.47 %) and high reliability (mean ICC = 0.83). Unadjusted SUV also performed well, with high reliability (ICC = 0.94) but also high variability (mean −1.24 %, SD 7.28 %). By comparison, the SUVR_C_ showed higher variability (mean −3.98 %, SD 7.07 %) and low reliability (ICC = 0.65).

**Conclusions:**

In this AD sample, we found that SUV-derived metrics of [^11^C]-PBR28 binding showed high stability over 12 weeks. These results compare favourably with studies reporting TRR of absolute quantification of [^11^C]-PBR28. Pending further validation of SUV-based measures of [^11^C]-PBR28, semi-quantitative methods of [^11^C]-PBR28 analysis may prove useful in longitudinal studies of AD.

**Electronic supplementary material:**

The online version of this article (doi:10.1186/s13550-016-0226-3) contains supplementary material, which is available to authorized users.

## Introduction

Activated microglia play an important role in the pathophysiology of Alzheimer’s disease (AD) [1]. Positron emission tomography (PET), using radioligands that bind to the mitochondrial 18 kDa translocator protein (TSPO), allows in vivo quantification of microglia in their activated state [2]. In comparisons with unaffected controls, TSPO binding has been shown to be significantly higher in AD and there has been interest in using second-generation TSPO ligands, including [^11^C]-PBR28, in longitudinal studies and clinical trials of immunomodulatory drugs [3].

Although [^11^C]-PBR28 is known to have a better signal-to-noise ratio and pharmacokinetic properties, compared to the first-generation TSPO tracer [^11^C]-R-PK11195, its use in longitudinal studies of AD is limited by several factors [4]. The absence of both a true reference region and an established pseudo-reference approach, such as the supervised clustering algorithm developed for [^11^C]-R-PK11195, means that most studies using [^11^C]-PBR28 require arterial cannulation [5]. This procedure adds cost, is invasive and can be uncomfortable. This limits the use of [^11^C]-PBR28 in studies with repeated measures and in AD patients, for whom tolerability and informed consent are significant issues. As an alternative to absolute quantification, three studies have recently demonstrated that a semi-quantitative, non-invasive approach using the standardised uptake value (SUV) or SUV ratio (SUVR) may be sensitive to changes in TSPO levels associated with neurodegenerative diseases including AD [6–8]. Although the SUV/SUVR do not provide details of compartmental binding, they are easy to derive, do not require arterial sampling and can reduce time spent in the scanner—appealing properties for use in patients with dementia. For these approaches to have utility for longitudinal studies, the test-retest reliability (TRR) needs to be established over a time period meaningful in clinical trials. In this study, a TRR analysis of the three published SUV-based methods for analysing [^11^C]-PBR28 data was conducted in a cohort of AD patients over a 12-week period.

## Materials and methods

### Patient recruitment

Participants were recruited from memory clinics in South London and Maudsley NHS Foundation Mental Health Trust. Inclusion and exclusion criteria were designed to reflect those of an on-going clinical trial of minocycline in patients with AD. Inclusion criteria included (i) age 55–95 years, (ii) diagnosis meeting the National Institute on Aging-Alzheimer’s Association (NIA/AA) criteria of possible or probable AD, (iii) standardised mini-mental state examination (sMMSE) >/=23 and (iv) consenting to participate [9, 10]. Exclusion criteria were (i) contraindication to administration of minocycline, (ii) presence of a variant of a polymorphism in the TSPO gene associated with low or medium tracer binding, (iii) contraindications to receiving a MRI scan and (vii) any medical conditions that might affect a person’s ability to tolerate the PET scan procedure [11]. Fourteen patients were screened for the study, and nine were excluded due to incompatible genotype. This study was ethically approved by the South Central Berkshire NRES Committee and was conducted according to the Declaration of Helsinki. All subjects gave written informed consent for participation.

### PET imaging procedure

Participants were scanned twice with a mean inter-scan time of 82 ± 10.2 days. Both scans were carried out on the same Siemens PET/CT camera at the Imanova Centre, London, and all patients were scanned at the same time of the day on both visits. [^11^C]-PBR28 was synthesised on site immediately prior to use according to local guidelines and regulations. For detailed description of synthesis and quality control procedures, please see Owen et al. (2014) [12]. [^11^C]-PBR28 was given as an intravenous infusion over 20 s. There were no significant differences between the doses injected at each scan (test = 339.0 ± 4.6 Mbq, retest = 350.8 ± 14.2 Mbq, *p* = 0.154). Dynamic image data were collected over 60 min and binned into 23 frames (durations: 8 × 15 s, 3 × 1 min, 5 × 2 min, 5 × 5 min, 2 × 10 min). Images were reconstructed using filtered back projection. A low-dose CT head scan was acquired for each subject for scatter and attenuation correction. Structural imaging (1.5TT1-weighted MRI) was carried out to exclude intracranial abnormalities and for co-registration.

### Genotyping

The SNP rs6971 was genotyped using TaqMan® SNP genotyping assays on a 7900HT sequence detection system (Applied Biosystems) and analysed using SDS software.

### Image analysis

Structural MRI scans were co-registered to PET images for tissue segmentation and region of interest (ROI) definition. A neuroanatomical atlas was co-registered onto each subject’s MRI data and PET image using a combination of Statistical Parametric Mapping 8 (http://www.fil.ion.ucl.ac.uk/spm) and FSL (http://www.fsl.fmrib.ox.ac.uk/fsl) functions, as implemented in MIAKAT™ (http://www.miakat.org/). The binding of [^11^C]-PBR28 in nine cortical and limbic ROIs was evaluated after applying a grey matter mask (see Table 1). These regions have previously been shown to be associated with increased TSPO signal in AD [3]. For bilateral regions, data from both hemispheres were analysed separately and then combined. The final 20-min window of the scan (40 to 60 min) was used to derive the SUV. Time-stability analysis for this time window is shown in Additional file 1. Unadjusted SUV in designated ROIs, SUV normalised to the whole brain (SUVR_WB_) and normalised to the cerebellum (SUVR_C_) were outcome measures, consistent with findings from previous reports as discussed above.

**Table 1:** Test-retest analysis of unadjusted SUV

	Mean test value	Mean retest	Mean regional difference % (SD)	Mean VAR % (SD)^a^	ICC	Sample size to detect 5 % change^b^	Sample size to detect 10 % change^b^
Frontal lobe	1.22	1.24	1.40 (6.62)	−1.22 (6.50)	0.96	21	7
Parietal lobe	1.17	1.19	2.33 (7.61)	−2.08 (7.49)	0.94	27	9
Temporal lobe	1.17	1.18	0.98 (7.34)	−0.76 (7.27)	0.93	25	8
Occipital lobe	1.21	1.22	0.87 (8.07)	−0.61 (8.10)	0.93	30	9
Hippocampus	1.06	1.08	2.01 (6.55)	−1.82 (6.48)	0.93	21	7
Parahippocampal cortex	1.15	1.18	2.91 (6.27)	−2.72 (6.08)	0.97	19	7
Posterior cingulate	1.31	1.35	3.27 (7.20)	−3.02 (7.03)	0.95	24	8
Amygdala	1.22	1.22	1.49 (6.01)	−1.34 (5.73)	0.98	18	7
Cerebellum	1.32	1.29	−1.81 (10.22)	2.28 (10.93)	0.87	45	14
Whole brain	1.13	1.14	1.35 (7.30)	−1.13 (7.22)	0.94	25	8
Mean (SD)	1.20 (0.08)	1.21 (0.08)	1.48 (7.32)	−1.24 (7.28)	0.94	26	8

*ICC* intraclass correlation coefficient

^a^VAR = absolute variability (%)

^b^Model parameters: two-tailed, power 0.9, alpha 0.05

### Statistical analysis

Test-retest reliability was determined by calculating the absolute variability between ‘test’ and ‘retest’ scans and intraclass correlation coefficient (ICC), using a two-way mixed model for consistency (class 3,1) in SPSS version 21 (www.spss.com). The mean regional difference (MRD) was calculated as follows: (Retest–Test/Test) * 100. The absolute variability (VAR) was calculated as follows: (2 * (Test–Retest))/(Test + Retest) * 100. Two-tailed sample size calculations required to detect a 5 and 10 % change in MRD were performed in using G*Power 3 software, with a power of 0.9 and probability of type I error of 0.05 using the SD of the MRD for each ROI.

## Results

### Sample characteristics

Five patients with high-affinity binding (HAB) genotype were included in the study (mean age 82.9 ± 4 years, four were male). Mean MMSE was 25.6 ± 1.3, and all patients were currently prescribed acetylcholinesterase inhibitors (AChEi). There were no significant differences in age or MMSE score between those included in the study and those excluded by genotype (mean age = 80.3 years ± 7.7 years, MMSE score = 25.8 ± 1.8).

### Test-retest analysis

The test-retest analysis of the three methods of SUV analysis is presented below—unadjusted SUV, SUVR_WB_ and SUVR_C_.

### Unadjusted SUV

The mean test-retest values, absolute variability and ICC for unadjusted SUV values are presented in Table 1. There was significant mean absolute variability (mean −1.24 %, SD 7.28 %) across ROIs. However, unadjusted SUV measurements were highly reliable as indicated by the high ICC values (mean ICC 0.94). From these data, a sample size of 26 would be required to detect a 5 % within-subject change in MRD (averaged across all ROIs), and a sample size of 8 would be required to detect a 10 % change.

### SUV relative to whole brain mean activity

The mean test-retest values, absolute variability and ICC for SUVR_WB_ are presented in Table 2. There was low absolute variability (mean −0.13 %, SD 2.47 %) across ROIs, and the SUVR_WB_ was highly reliable (mean ICC 0.83). Consequently, a sample size of 7 would be required to detect a 5 % within-subject change in MRD (averaged across all ROIs), and a sample size of 4 would be required to detect a 10 % change.

**Table 2:** Test-retest analysis of SUV relative to whole brain mean activity (SUVR_WB_)

	Mean test value	Mean retest value	Mean regional difference % (SD)	Mean VAR% (SD)^a^	ICC	Sample size to detect 5 % change^b^	Sample size to detect 10 % change^b^
Frontal lobe	1.08	1.08	0.096 (1.42)	−0.09 (1.41)	0.96	4	3
Parietal lobe	1.03	1.04	0.96 (0.81)	−0.95 (0.80)	0.98	3	3
Temporal lobe	1.04	1.03	−0.36 (0.73)	0.37 (0.73)	0.90	3	3
Occipital lobe	1.07	1.06	−0.51 (1.35)	0.52 (1.37)	0.91	4	3
Hippocampus	0.94	0.95	0.71 (2.09)	−0.69 (2.06)	0.97	5	3
Parahippocampus	1.01	1.03	1.64 (3.07)	−1.59 (2.98)	0.48	7	4
Posterior cingulate	1.15	1.17	1.92 (1.36)	−1.89 (1.33)	0.98	4	3
Amygdala	1.07	1.07	0.35 (5.79)	−0.21 (5.77)	0.69	17	6
Cerebellum	1.17	1.13	−3.24 (5.40)	3.42 (5.74)	0.63	15	6
Mean (SD)	1.06 (0.07)	1.06 (0.06)	0.17 (2.45)	−0.13 (2.47)	0.83	7	4

*ICC* intraclass correlation coefficient

^a^VAR = absolute variability (%)

^b^Model parameters: two-tailed, power 0.9, alpha 0.05

### SUV normalised to cerebellar activity

The mean test-retest values, absolute variability and ICC for SUVR_C_ are presented in Table 3. There was considerable absolute variability (mean −3.98 %, SD 7.07 %) and SUVR_C_ showed poor reliability (mean ICC 0.65). Overall, a sample size of 25 would be required to detect a 5 % within-subject change in MRD (averaged across all ROIs), and a sample size of 8 would be required to detect a 10 % change. Of the three measures, the SUVR_C_ performed most poorly, due to high levels of variability in binding in the cerebellar grey matter reference region.

**Table 3:** Test-retest analysis of SUV relative to cerebellar activity (SUVR_C_)

	Mean Test Value	Mean Retest Value	Mean regional difference % (SD)	Mean VAR % (SD)^a^	ICC	Sample size to detect 5 % change^b^	Sample size to detect 10 % change^b^
Frontal lobe	0.93	0.96	3.78 (7.63)	−3.50 (7.06)	0.69	27	9
Parietal lobe	0.89	0.93	4.62 (6.37)	−4.37 (5.94)	0.70	20	7
Temporal lobe	0.89	0.92	3.23 (5.87)	−3.05 (5.22)	0.68	17	6
Occipital lobe	0.92	0.94	3.04 (4.98)	−2.90 (4.75)	0.71	13	5
Hippocampus	0.81	0.84	4.44 (8.18)	−4.11 (7.54)	0.77	31	10
Parahippocampus	0.87	0.92	5.46 (9.51)	−5.00 (8.59)	0.38	40	12
Posterior cingulate	0.99	1.05	5.65 (7.25)	−5.31 (6.65)	0.79	25	8
Amygdala	0.92	0.95	4.18 (11.53)	−3.62 (10.51)	0.47	25	8
Mean (SD)	0.90 (0.05)	0.94 (0.06)	4.30 (7.66)	−3.98 (7.07)	0.65	25	8

*ICC* intraclass correlation coefficient

^a^VAR = absolute variability (%)

^b^Model parameters: two tailed, power 0.9, alpha 0.05

## Discussion

Of the three SUV measures present in this test-retest analysis, the SUV normalised to whole brain mean activity performed most strongly. The unadjusted SUV showed higher variability, and the SUVR_C_ showed both higher temporal variability and lower reliability. Lower reliability in SUVR methods, as measured by ICC, was most apparent in smaller regions with lower signal to noise ratios, such as the parahippocampal gyrus, which is affected early in the AD process.

Compared to published data on the TRR of [^11^C]-PBR28, the SUV methods presented here perform well [13, 14]. Park et al. reported that, over a 1.4-week period, in healthy controls and patients with multiple sclerosis, the SDs of test-retest variability of *V*_T_ and *V*_T_/*f*_p_ (derived using a multi-linear analysis) varied from 9 to 11 % and 7 to 14 %, respectively [13]. Collste et al. [14] reported that the mean absolute variability in *V*_T_ in the grey matter was 18.3 ± 12.7 %, with ICC values from 0.90 to 0.94, and that, using a parametric modelling approach, variability was 17.8 ± 12.7 %. Participants were imaged either on the same day or 1 day apart. By comparison, all three SUV measures in our study performed well, over a much longer time period. These results also compare favourably with TRR analysis of the first-generation TSPO ligand [^11^C]-R-PK11195 [5, 15].

These findings should be viewed as a preliminary indicator that SUV-based methods may be suitable for use in longitudinal studies in AD. Our findings should be confirmed in larger samples, which could also include medium-affinity binders to assess the impact of genotype on longitudinal variability. Further work is also needed to confirm the relationship between SUV measures and measures of specific binding. Although there are concerns about the non-specific binding profile of [^11^C]-PBR28, work by Lyoo et al. suggests that SUVR_C_ correlates well with absolute quantification, but more data on this is required [8, 16]. This validation is also required for alternative SUV-derived methods, and this could be easily achieved through retrospective analysis of existing data sets. Although SUV and SUVR_WB_ appear to have better test-retest reliability, other factors such as sensitivity to disease states may vary between methods and should be considered when choosing a quantification method.

In conclusion, given the caveats listed above, this TRR analysis of SUV-derived measures of [^11^C]-PBR28 binding in AD suggests that non-invasive semi-quantitative approaches are stable and reliable over significant periods of time.

## Additional file

Additional file 1: Time-stability analysis for 40-60 mins time window. (DOCX 125 kb)
