# Erratum to: Test-retest analysis of a non-invasive method of quantifying [(11)C]-PBR28 binding in Alzheimer's disease

**DOI:** 10.1186/s13550-017-0256-5

**Published:** 2017-02-14

**Authors:** Akshay Nair, Mattia Veronese, Xiaohui Xu, Charles Curtis, Federico Turkheimer, Robert Howard, Suzanne Reeves

**Affiliations:** 1 UCL Huntington’s Disease Research Group, 2nd floor, Russell Square House, 10-12 Russell Square, London, WC1B 5EH UK; 2 Centre for Neuroimaging Sciences, IoPPN, King’s College London, Box PO89De Crespigny Park, London, SE5 8AF UK; 3 MRC Social, Genetic and Developmental Centre, Institute of Psychiatry, Psychology & Neuroscience, King’s College London, De Crespigny Park, Denmark Hill, London, SE5 8EF UK; 4 NIHR Biomedical Research Centre for Mental Health, Maudsley Hospital and Institute of Psychiatry, Psychology & Neuroscience, King’s College London, London, SE5 8AF UK; 5 Division of Psychiatry, University College London, 6th Floor, Wings A and B, Maple House, 149 Tottenham Court Road, London, W1T 7NF UK

## Erratum

Following publication of the original article [1] the authors contacted the team with the request that some additional information be added to the article. Please see below for details:

**Additional Table: absolute variability**

Variability (VAR) presented in the main article represents **mean variability** between test and retest, not absolute variability as stated. The authors would like to apologise for this error. The absolute variability is presented in the table below has been calculated in the following manner:

$$ \mathbf{Absolute}\ \mathbf{variability} = \left(\mathbf{2}*\left(\left|\mathbf{Test}\ \hbox{--}\ \mathbf{Retest}\right|\right)\right)\ /\ \left(\mathbf{Test} + \mathbf{Retest}\right)\Big)\ *\ \mathbf{100} $$

**Table:** 

	Raw SUV	SUV_WB_	SUV_C_
Frontal Lobe	6.05	1.01	4.36
	(2.45)	(0.67)	(4.84)
Parietal Lobe	6.69	0.97	5.15
	(2.33)	(0.77)	(5.10)
Temporal Lobe	5.92	0.61	3.78
	(3.13)	(0.49)	(4.92)
Occipital Lobe	6.81	1.07	3.87
	(2.83)	(0.88)	(3.79)
Hippocampus	5.82	1.57	5.35
	(1.95)	(1.34)	(6.50)
Parahippocam-pal cortex	5.05	2.07	5.77
	(3.78)	(2.58)	(7.96)
Posterior Cingulate	6.58	1.89	5.92
	(2.60)	(1.33)	(5.97)
Amygdala	3.45	4.31	6.54
	(4.50)	(3.17)	(8.56)
Cerebellum	7.18	4.18	
	(7.84)	(5.07)	
Whole Brain	6.09		
	(2.73)		
Mean (SD)	5.96	1.96	5.09
	(3.41)	(1.81)	(5.95)

These analyses allow for easier comparison with other studies however do no alter the findings or discussion of the paper – SUV normalised to whole brain (SUV_WB_) shows the lowest variability in our cohort, with increased variability found in the unadjusted SUV and the SUV normalised to cerebellum. As compared to other test-retest studies discussed in the paper, the variability for SUV based methods remains favourable in this pilot cohort. These results should be validated in larger studies.
