# Supplementary material for: Test-retest analysis of a non-invasive method of quantifying [11C]-PBR28 binding in Alzheimer’s disease
Source: EJNMMI Res. 2016 Sep 27;6:72. doi: 10.1186/s13550-016-0226-3 (PMC5039146; doi:10.1186/s13550-016-0226-3)
Supplement: Additional file 1: — Time-stability analysis for 40-60 mins time window. (DOCX 125 kb) [file 13550_2016_226_MOESM1_ESM.docx]

Manuscript ref no.: EJRE-D-16-00074R1

Supplementary data:

Date: 12^th^ September 2016

Time stability analysis:

Due to the nature of the patient population, tolerability was a concern and therefore the maximum scanning time was 60 minutes and no kinetic modelling was possible. Nevertheless, SUVR estimates normalised to whole brain were shown to be stable over time (absolute mean relative difference (MRD) <2%, MRD = (SUV(X) - SUV(40-60))/SUV(40-60) while SUVR estimates normalised to cerebellum showed higher variability, particularly at early time points. A time stability analysis is shown in the figure below and included as supplementary data.


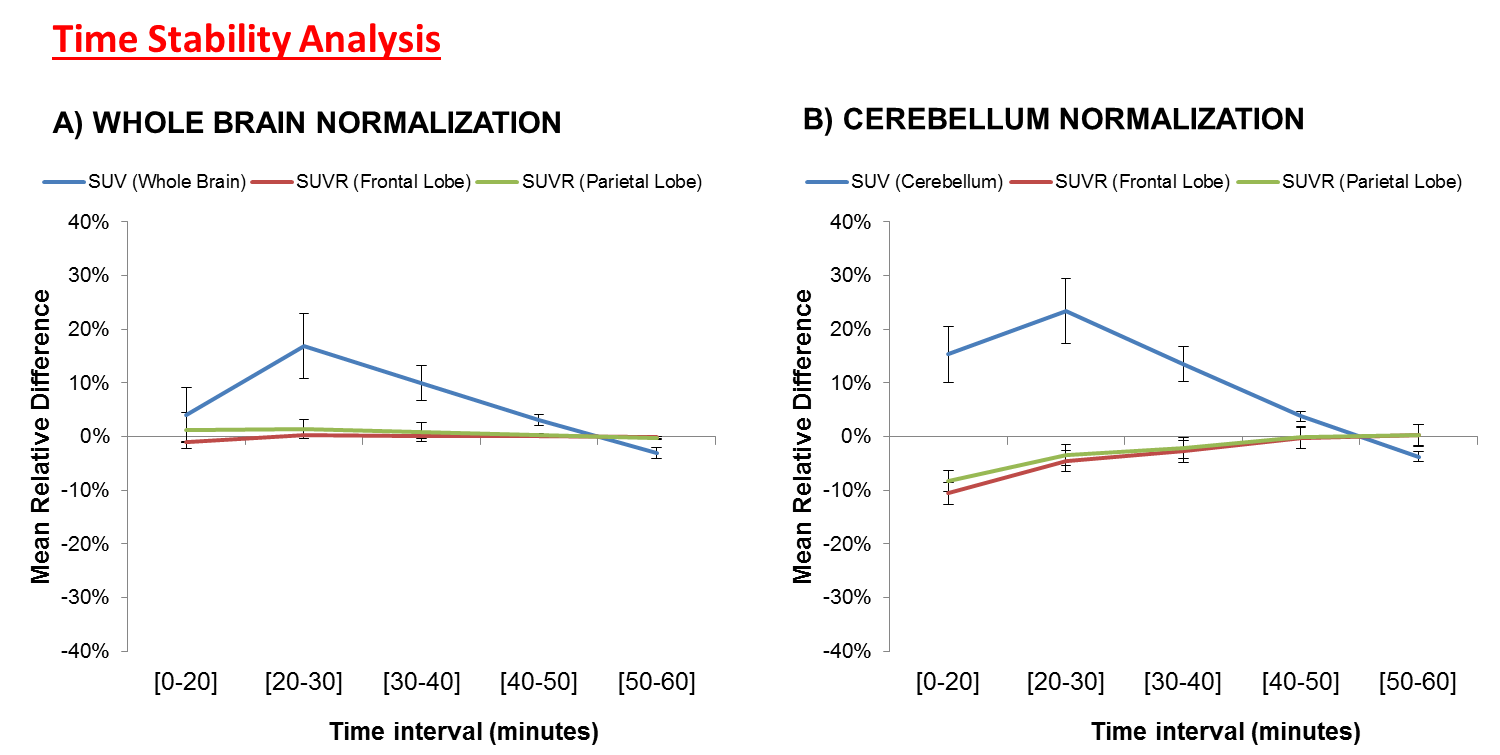


*Figure (1): SUV and SUVR time stability analysis, using whole brain and cerebellum as normalisation regions (panel A and B respectively). The mean relative difference to SUV and SUVR calculated at 40-60 min post-injection interval are shown as function of time.*
